# Prestimulus Alpha Phase Modulates Visual Temporal Integration

**DOI:** 10.1523/ENEURO.0471-23.2024

**Published:** 2024-09-12

**Authors:** Michelle Johannknecht, Alfons Schnitzler, Joachim Lange

**Affiliations:** Institute of Clinical Neuroscience and Medical Psychology, Medical Faculty and University Hospital Düsseldorf, Heinrich Heine University Düsseldorf, Düsseldorf 40225, Germany

**Keywords:** alpha, MEG, oscillation, phase, temporal integration, vision

## Abstract

When presented shortly after another, discrete pictures are naturally perceived as continuous. The neuronal mechanism underlying such continuous or discrete perception is not well understood. While continuous alpha oscillations are a candidate for orchestrating such neuronal mechanisms, recent evidence is mixed. In this study, we investigated the influence of prestimulus alpha oscillation on visual temporal perception. Specifically, we were interested in whether prestimulus alpha phase modulates neuronal and perceptual processes underlying discrete or continuous perception. Participants had to report the location of a missing object in a visual temporal integration task, while simultaneously MEG data were recorded. Using source reconstruction, we evaluated local phase effects by contrasting phase angle values between correctly and incorrectly integrated trials. Our results show a phase opposition cluster between −0.8 and −0.5 s (relative to stimulus presentation) and between 6 and 20 Hz. These momentary phase angle values were correlated with behavioral performance and event-related potential amplitude. There was no evidence that frequency defined a window of temporal integration.

## Significance Statement

In light of the current debate whether visual perception is a rhythmic or discrete process, we give new insight into this debate. We investigated the neural mechanisms defining potential rhythmic perception. In a visual temporal integration task, we were able to show that the processing of incoming information depended on the phase of neuronal alpha/beta oscillations. Our data support the idea that the phase of prestimulus alpha oscillation modulates poststimulus visual processing by defining good and less good phases for early visual processes. We were not able to show that prestimulus alpha oscillation defines windows where two visual stimuli are integrated into one single event.

## Introduction

Although our everyday experience implies that perception is a seamless process, the human perceptual system is limited to accurately detect and process incoming information. These limitations affect not only the spatial but also the temporal resolution of perception. For example, a series of discrete stimuli will be perceived as a continuous, seamless flow of information, if the presentation duration is faster than the temporal resolution of the visual system—like in movies.

Research in the last decades has highlighted the functional role of neuronal oscillations for perception. Especially prestimulus alpha oscillations have been shown to be a relevant factor for perception near threshold. For example, recent studies demonstrated that the power of alpha oscillations influences perception in near-threshold detection tasks (van Dijk et al., 2008; Lange et al., 2013; Baumgarten et al., 2016; Limbach and Corballis, 2016; Iemi et al., 2017; Benwell et al., 2022).

In addition, evidence showed that the momentary phase of prestimulus oscillations influences the detectability of stimuli in near-threshold detection tasks (Busch et al., 2009; Mathewson et al., 2009; Busch and VanRullen, 2010; Dugue et al., 2011; Landau and Fries, 2012). While most of the evidence for the potential role of phase for perception stems from detection tasks, few studies also reported an influence of phase on temporal perception (Baumgarten et al., 2015; Milton and Pleydell-Pearce, 2016; Ikumi et al., 2019).

Despite the accumulating evidence for a functional role of alpha phase in visual perception, there are still unresolved questions and remaining concerns. For instance, while some studies reported an influence of alpha phase on perception, other studies reported null results (summarized by Keitel et al., 2022). Moreover, inconsistencies in the reported frequency ranges in similar tasks raise the question of the validity of these findings (Merholz et al., 2022). Finally, the functional role of prestimulus phase remains unclear. Prestimulus phase might modulate sensory processing by modulating poststimulus-evoked potentials (Busch and VanRullen, 2010; Milton and Pleydell-Pearce, 2016). Other studies propose that the phase effects may indicate that specific frequency bands are relevant for perceptual integration or segregation of uni- or multimodal stimuli (Baumgarten et al., 2015; Wutz et al., 2018; Ikumi et al., 2019).

While most studies focused on the role of phase for detection, few studies investigated the role of phase for temporal integration of visual stimuli. Wutz et al. (2016) used the same task as we used in the present study to investigate visual temporal integration or segregation, with a strong focus on the neural activity in the theta band. They reported phase differences in the theta band depending on the task (integration or segregation) and participants' responses (correct or incorrect; Wutz et al., 2016). In a follow-up study, the authors focused on the functional role of frequency for visual integration and segregation (Wutz et al., 2018). In addition, Milton and Pleydell-Pearce (2016) found that prestimulus phase correlated with performance in a temporal simultaneity task. In the present study, we expand these findings by determining whether the prestimulus phase influences temporal integration mechanisms by modulating early poststimulus sensory processing or whether the frequency of prestimulus phase effects determines temporal integration windows. We want to deepen the understanding of the neural mechanisms of temporal integration by looking into the momentary prestimulus phase in a broad spectrum of frequencies. First, we want to investigate how the prestimulus phase influences behavioral performance. Further, in addition to most other studies, we want to investigate whether putative prestimulus phase effects mediate poststimulus neural processing or whether prestimulus phase effects support the hypothesis of temporal windows of integration. To investigate these hypotheses, participants performed a visual temporal integration study (Wutz et al., 2016), while we simultaneously recorded their neural activity using MEG. We analyzed whether the phase of neuronal oscillations correlated with the successful integration of the visual stimuli and poststimulus sensory processing.

## Materials and Methods

### Participant and ethical information

We recruited 25 participants [17 female; mean age 25.4 ± 5.1 years (SD)] for this study. We chose the number of participants based on previous papers using the same task (Wutz et al., 2016, 2018). The participants provided written informed consent in line with the Declaration of Helsinki, and the Ethical Committee of the Medical Faculty, Heinrich Heine University, approved the study. No participant reported to have any neurological or psychological disorder. All participants had normal or corrected-to-normal vision. Participants received 10€ per hour for participation.

### Stimuli and task

**Figure 1.**
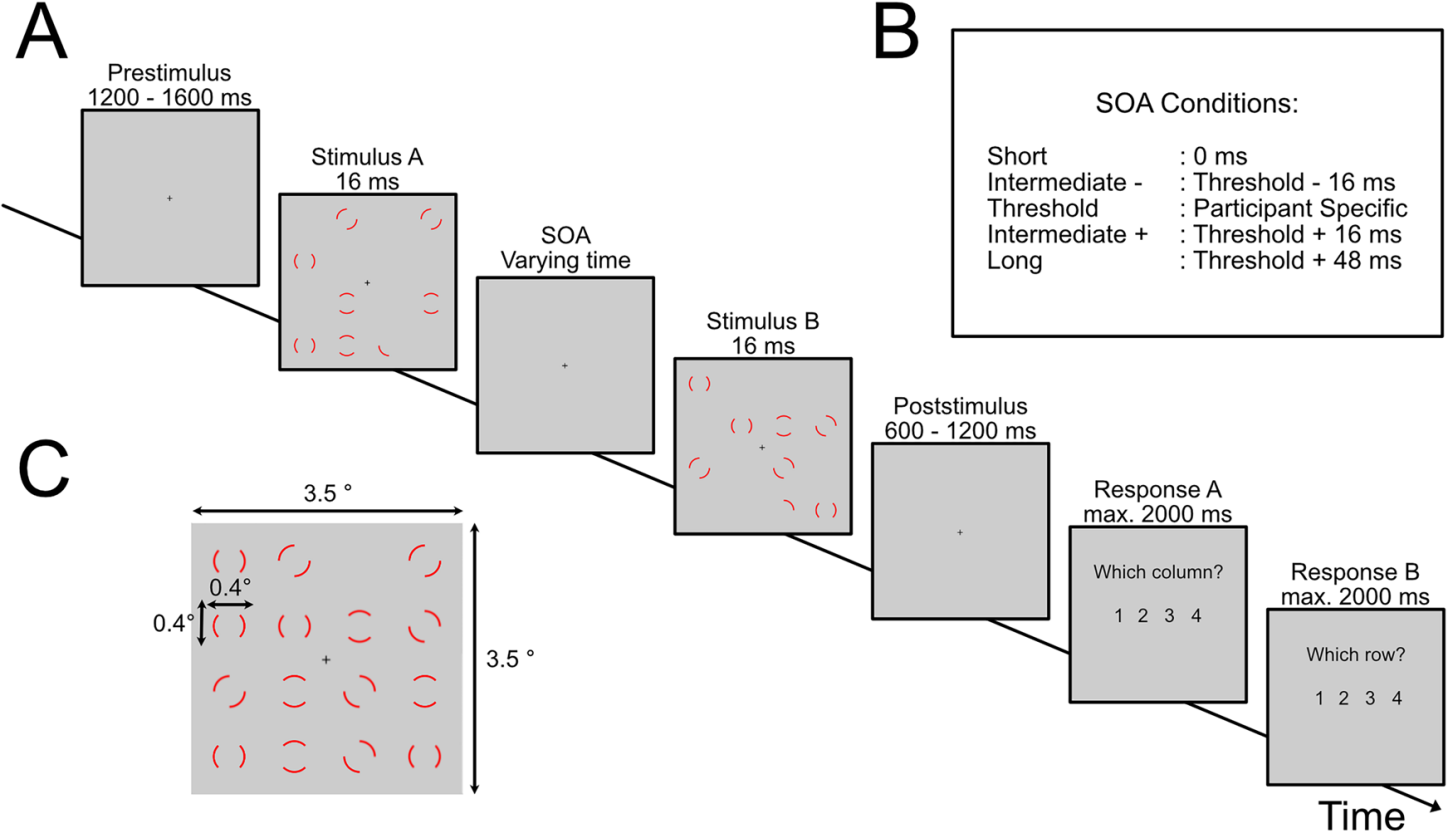
Paradigm. ***A***, The experiment started with a prestimulus phase (randomly between 1,200 and 1,600 ms) where only a fixation cross was presented. Afterward, the first stimulus was presented for 16 ms, followed by a stimulus offset asynchrony (SOA) and the second stimulus. After a poststimulus period (randomly between 600 and 1,200 ms), participants reported the position of the empty location. ***B***, The different SOA conditions used in the experiment. The threshold SOA was individually determined to achieve 50% accuracy. ***C***, An example of a full integration of the two stimuli.

We adapted the stimuli and task design from Wutz et al. (2016). Each trial started with a randomized prestimulus period between 1,200 and 1,600 ms with a central fixation cross, followed by the presentation of the stimulus (Fig. 1). A stimulus consisted of two images presented for 16 ms each. Both images were separated by individual stimulus onset asynchronies (SOAs, see below for details on the SOA). Each image showed seven full annuli and one-half of an annulus presented on pseudorandom positions on a 4 × 4 grid. When both images are integrated, annuli fill out each position, except one. A poststimulus period followed the stimuli, with a random duration between 600 and 1,200 ms with only a fixation cross. Finally, an instruction text appeared which prompted participants' responses. Participants' task was to report the empty position. Importantly, participants could report the position only if they temporally integrated both images. Participants reported the position of the missing element by responding twice: The first response was to report the number of the row of the missing element, and the second response was to report the column number within the 4 × 4 grid. Participants responded with their right hand via button press. When they responded too early (before presentation of the instructions) or too late (>2,000 ms after presentation of instructions), they received feedback accordingly, and the trials were appended to the end of the block.

We projected the stimuli on a translucent screen using a projector located outside the magnetically shielded room (Panasonic, PT-DW700E; 60 Hz refresh rate) and a mirror system. The screen was 140 cm away from the participant. Grid dimensions were 8 × 8 cm (i.e., visual angle of 3.5 × 3.5°; each annulus was 1 × 1 cm in size; annuli were evenly spaced on the grid). A training session of ∼2–5 min preceded the experiment.

Before the main experiment, we used a staircase method to measure the performance threshold of 50% performance accuracy. The staircase started with a fixed SOA of 26 ms. The SOA increased when participants answered twice in a row correctly or decreased when they answered twice incorrectly. The step size of the increase/decrease was 16 ms (e.g., one frame). When performance was stable at 50% accuracy over the last 20 trials (i.e., the variance of SOAs was <0.5), the staircase was terminated, and the current SOA was taken as the threshold SOA. The staircase was interleaved with SOAs randomly picked from the predefined SOA distribution (starting at 0 ms and increasing in steps from 16 to 144 ms). These random SOAs were not included in the computation of the threshold SOA. The threshold SOA is the temporal offset between the two images the participant needed to integrate both images in 50% of trials. We used additional SOA conditions [0 ms (i.e., both images presented directly after each other), threshold SOA ± 16 ms, threshold SOA + 48 ms] to monitor participants' behavior (Baumgarten et al., 2015, 2017). One block consisted of two repetitions of the long (threshold SOA + 48 ms) and short SOA (0 ms) each, four repetitions for each of the intermediate conditions, and fifteen repetitions of the threshold SOA, all stimuli presented in pseudorandomized order. The entire experiment consisted of 15 blocks and lasted between 30 and 45 min. After every 100 trials, the participant could take a self-paced break. We used the software Presentation (Neurobehavioral Systems) to control the experiment.

### MEG recording

During the experiment, we recorded the electromagnetic signal of the brain using a 306-channel magnetoencephalography (MEG) system (MEGIN), with a sampling rate of 1,000 Hz. The electrooculogram (EOG) was simultaneously recorded by placing electrodes above and below the left eye and on the right and left temples. The head position inside the MEG was coregistered using four head position indicator (HPI) coils. We placed the coils behind the left and right ears and on the right and left foreheads. We digitized the coil positions, anatomical landmarks (nasion, left and right periauricular points), and ∼50–100 additional points from the head using the Polhemus digitizer (Fastrak, Polhemus).

### MRI recording

For each subject, we recorded a structural magnetic resonance image, with a 3 T MRI scanner (Siemens). Afterward, MEG data were aligned offline with the MRI using anatomical landmarks (nasion, left and right preauricular points).

### Behavioral analysis

We analyzed the behavioral data on a group level by calculating the fractional accuracy for each SOA condition and subject. We averaged the individual fractional accuracies across participants. To test for the statistical difference in accuracy per SOA, we performed a one-way ANOVA and post hoc Tukey–Kramer tests for pairwise comparisons between SOAs. See also Table S1 for an overview of the statistical tests performed.

### Preprocessing of MEG data

For the analysis of MEG data, we used MATLAB (R2019b) and the analysis toolbox FieldTrip (Version 20210825; Oostenveld et al., 2011). First, we divided the continuous MEG data into trials starting with the presentation of the fixation cross and ending with the presentation of the response instructions. We removed jump artifacts, eye movement, and muscle movement, using a semiautomatic approach implemented in FieldTrip. We applied band-stop filters from 49 to 51, 99 to 101, and 149 to 151 Hz, to remove the power line and a bandpass filter between 2 and 200 Hz to the data. Next, we visually inspected the data to remove noisy channels and trials. Finally, we used independent component analysis (ICA) to remove undetected noise or artifacts. To speed up ICA, we resampled the data to 150 Hz.

### Source projection of MEG data

Source grid models were computed by applying a regular-spaced 3D grid with 5 mm resolution to the Montreal Neurological Institute (MNI) template brain provided by the FieldTrip toolbox in MATLAB (R2019b). We computed individual grids by nonlinearly warping the individual structural MRI on the MNI MRI, after which we applied the inverse of this warp to the MNI template grid, which resulted in the individual warped virtual sensor grid.

Next, we calculated spatial filters in the time domain by computing a lead field matrix for the reconstructed warped visual sensor grid (Nolte, 2003) and using data between −1 and −0.5 s. We calculated spatial filters using the linear constrained minimum variance beamforming approach (Van Veen, 1997) and projected the sensor time-series MEG data through these filters. This projection resulted in time-series data for each grid point. We restricted from here further analyses to regions of interest (ROI), defined by the AAL atlas (Tzourio-Mazoyer et al., 2002) implemented in FieldTrip. We expected to see effects in early visual areas and thus restricted the ROI to these areas. We included the following regions (left and right hemisphere): calcarine fissure, precuneus, lingual gyrus, cuneus, superior occipital lobe, middle occipital lobe, inferior occipital lobe, superior parietal gyrus, and inferior parietal gyrus.

### Calculating phase opposition sum and surrogate data

To analyze whether the phase of neuronal oscillations influences temporal integration, we included only threshold SOA trials. We sorted the trials into three different groups: “correct” (including only trials where the coordinates of the missing element were correctly reported), “incorrect” (trials where the coordinates were not correctly reported), and the combination of correct and incorrect, which will be labeled “all.”

We analyzed phase differences between correct and incorrect trials using the phase opposite sum (POS, VanRullen, 2016a). To this end, we calculated for each grid point and trial a fast Fourier transformation (FFT) for frequencies between 2 and 30 Hz in steps of 2 Hz. The time window of interest was the prestimulus period between −1 and 0 s (onset of stimulus). We used a sliding window of 0.3 s length and moved in steps of 0.05 s. Prior to FFT, we multiplied the data with a single Hanning taper. Next, we calculated for each condition the intertrial coherence (ITC), which is a measure of phase consistency across trials (VanRullen, 2016a).

$$\mathrm{IT}C_{\mathrm{all}}=\;\left|\underset{\left[i\;=\;1:n\right]}{\sum }\;\omega_{i}/|\omega_{i}|\right|/n,$$

$$\mathrm{IT}C_{\mathrm{correct}}=\;\left|\underset{\left[i\;\in \;n_{\mathrm{correct}}\right]}{\sum }\;\omega_{i}/|\omega_{i}|\right|/n_{\mathrm{correct}},$$

$$\mathrm{IT}C_{\mathrm{incorrect}}=\;\left|\underset{\left[i\;\in \;n_{\mathrm{incorrect}}\right]}{\sum }\;\omega_{i}/|\omega_{i}|\right|/n_{\mathrm{incorrect}}.$$

*N* refers to the number of trials in each group, and *ω* refers to the angle at trial *i*. Using the ITC values, we calculated the phase opposition sum (POS).

$$\mathrm{POS}=\mathrm{IT}C_{\mathrm{correct}}+\mathrm{IT}C_{i\mathrm{correct}}-2\mathrm{IT}C_{\mathrm{all}}.$$

The POS value was calculated for each time–frequency grid point sample independently. POS values are bound between zero and two, zero indicating that the phase values between correct and incorrect are identical, and positive values indicate that phase values are different between correct and incorrect. Since trial number biases ITC, we picked randomly a subset of trials from the group with the higher trial count to equal the number of trials with the group containing fewer trials and then computed ITC and POS values. The average trial number was 87.4 (±34.4 STD) for the correct trials and 81.2 (±25.1 STD) for the incorrect trials. We repeated this step 100 times, resulting in 100 POS values per time–frequency grid point sample. We calculated the median of POS values per subject and time–frequency grid point sample.

These observed POS values were statistically tested against a null distribution of surrogate data. We created surrogate data by shuffling the trials from groups correct and incorrect, while accounting for an equal trial count. We selected 100 times random trials and calculated the mean POS values. POS values were calculated as described above. We calculated for each participant 100 mean POS values for each time–frequency grid point.

To test for group-level effects of the POS effect, we used a cluster-based permutation approach (Oostenveld et al., 2011). We first normalized the individual POS values by subtracting the mean and dividing them by the standard deviation of the surrogate data. Then, we statistically compared observed and surrogate data across subjects by means of a dependent-sample *t* test. We used the Monte Carlo method with 1,000 repetitions. This step was done independently for each time–frequency grid point sample. Next, we combined data points with a *p*-value of <0.05, which were adjacent in time, frequency, and space to a cluster. We predefined a neighbor structure for the data, which contains the positions of the channel and its relative neighbors, and restricted the neighbors to be a maximum of 0.6 cm away. The minimum cluster size was set to 2.

To obtain the surrogate cluster distribution, we calculated *p*-values for each time–frequency grid point and repetition, by testing every surrogate value against the other surrogate values of that time–frequency grid point. We defined clusters with the same parameters as described for the observed data. This process was repeated 1,000 times, always selecting the largest surrogate cluster found. This resulting surrogate cluster distribution was used to test the observed data against it (Table S1).

In an additional post hoc analysis, we investigated if power had a systematic influence on our reported phase effects (see Results). We median split the threshold SOA trials into low- and high-power trials and calculated the POS values as described above. We averaged the POS values for each participant over the channel–time–frequency points where we found phase effects. We applied a *t* test to test for systematic differences between phase values between high- and low-power trials.

### Phase-dependent behavioral performance

The POS analysis provides information on whether the phase of neuronal oscillations differs between correct and incorrect trials. For a more detailed understanding of the relationship between prestimulus phase and performance accuracy, we calculated the participants' fractal accuracy for different phase bins. To this end, we first determined in each participant the time–frequency sample with the highest POS value within the grid points belonging to the significant cluster in the POS analysis (Baumgarten et al., 2015). For this time–frequency sample, we computed the momentary phase for each threshold SOA trial (see above for the parameters). Next, we binned the phase values from −π to +π in steps of (1/3)π. For each of these six phase bins, we calculated the individual performance averaged across all trials. We normalized individual performance by subtracting the mean of all bins from each bin. Next, we aligned the bins so that the bin with the highest performance was aligned to Bin 0. Finally, we averaged data per bin across participants. To test for statistical significance, we performed a one-way repeated ANOVA (Table 1), while Bin 0 was excluded from the statistics, and post hoc Tukey–Kramer tests for pairwise comparisons between phase bins. The average trial count per bin was 27.7 (±7.7 SD) for Bin 1, 28.2 (±8.2 SD) for Bin 2, 26.0 (±7.4 SD) for Bin 3, 29.6 (±8.6 SD) for Bin 4, 27.2 (±7.4 SD) for Bin 5, and 28.5 (±6.7 SD) for Bin 6.

**Table 1.** Statistical test overview

Data structure	Type of test	Power
Behavioral data (non-normal distributed)	One-way ANOVA	CI 95%
Phase opposition sum values, normalized (normal distribution)	Cluster-based permutation testing	CI 95%
Phase binned behavioural data (non-normal distributed)	One-way repeated ANOVA	CL 95%
Phase binned ERP data (non-normal distributed)	One-way repeated ANOVA	CL 95%
Peak frequency values (non-normal distributed)	Pearson’s correlation	CL 95%

The table shows for which data structure, we performed which test and the confidence intervals.

### Phase-dependent ERF

We investigated whether prestimulus phase affects visual stimulus processing. To this end, we used an early visual component of the event-related field (ERF)—the N170/N1 component—as a measure of stimulus processing (Mazaheri and Picton, 2005; Han et al., 2015). The individual latency of the component was based on a data-driven approach. We took the first component in our data, which had a negative deflection at ∼170 ms. Hence, we called it the N170 component. First, we determined 100 grid points showing the highest ERF values. To this end, we calculated for each grid point *t* values for the ERF by applying a dependent-sample *t* test between poststimulus ERF (0–0.3 s) and prestimulus ERF (averaged across −0.8 to −0.5 s) and selected 100 grid points showing the highest absolute *t* value averaged across 170 ms ± 25 ms. The N170 component was therefore based on an average of time points, and this was done to account for varying time points between subjects. Next, from these 100 selected channels, we computed for each participant the momentary phase for the threshold SOA trials. We binned the phase as described above and recalculated the ERF *t* values, this time only for the trials within each bin. We averaged the ERF *t* value across all 100 grid points for each participant and aligned the highest value to Bin 0. Finally, we averaged the *t* values per bin over participants and performed a one-way repeated measure ANOVA, excluding bins with Phase 0, and post hoc Tukey–Kramer tests for pairwise comparisons between phase bins (Table S1). The average trial count for the six bins was 26.5 (±8.0 SD) for Bin 1, 27.5 (±6.3 SD) for Bin 2, 29.1 (±8.6 SD) for Bin 3, 30.2 (±8.3 SD) for Bin 4, 26.3 (±7.5 SD) for Bin 5, and 27.5 (±7.0 SD) for Bin 6. We additionally visualized the averaged ERF time course for three different phase bins. The ERF data were first phase binned with the same binning method as described above and then aligned to phase Bin 0. The difference is that the data was not averaged over the N170 component, but the complete time course was used. Additionally, we tested whether the phase binning of the behavioral analysis (see above) leads to similar results. This would indicate that the phase, relevant to the behavioral effect, also directly affects the ERF amplitude. Therefore, we sorted the ERF trials based on the phase values of the behavioral data. Then the ERF values were binned and aligned to phase Bin 0.

### Correlation of peak frequency with threshold SOA

Previous studies reported a correlation between participants' perceptual temporal resolution and the peak frequency of neuronal oscillations (Samaha et al., 2015). Based on these findings, we tested if the individual peak frequency correlates with the SOA derived from the staircase and the theoretical fitted SOA. For the theoretically fitted SOA, we fitted a sigmoid function onto the individual behavioral data using the Palamedes toolbox for MATLAB (Prins and Kingdom, 2018). From the fitted function, we computed the SOA for which the participant reached 50% correct responses. This SOA, we call the fitted threshold SOA. We used this second approach to account for potential noise in the initial threshold SOA estimation. We estimated the peak frequency for each participant not based on power values but on the highest POS value. To this end, we selected within the significant cluster in the POS analysis the frequency of the highest POS value per participant (using the MATLAB findpeaks.m function). Finally, we correlated (Pearson's correlation) the estimated individual peak frequencies with the individual threshold SOA estimated during the staircase and with the fitted SOA (fitted SOA; Table S1). Additionally, we fitted a general linear model onto the data for visualization. We are aware that this is not a conventional method to investigate the influence of peak frequency on the temporal integration window (Cecere et al., 2015; Samaha and Postle, 2015; Baumgarten et al., 2017; Tarasi and Romei, 2024). Therefore, we ran a complementary analysis to detect power peaks, correlated them with the staircase and fitted threshold SOA. The prestimulus power spectrum was calculated using a single Hanning window with a frequency resolution of 2 Hz, between 2 and 30 Hz. With the FOOOF toolbox (Version 1.1.0; Donoghue et al., 2020), we corrected for the 1/*f* component. We determined for each virtual channel the frequency with the highest power using findpeaks.m. We calculated the average peak frequency for the cluster channels, for each subject, and correlated those with the staircase and fitted SOA.

### Code accessibility

The analysis was run on Linux using MATLAB (R2019b), using a 40-core computer with 120 GB RAM, operating system is Debian 10 (Buster). Due to their large size, the data files will be made available upon request. Analysis scripts are made unrestrictedly available and can be found following this link: https://osf.io/ah3gf/?view_only=0d058e3560cf4eeeafdee337f6ab8c22.

## Results

Participants performed a visual temporal discrimination task in which they were presented with two grids partially filled with annuli separated by SOAs of varying duration. If both grids were integrated, only one position was not filled with an annulus. Participants' task was to report by button press whether they detected the empty position, i.e., whether they were able to temporally integrate both stimuli.

### Behavioral results

**Figure 2.**
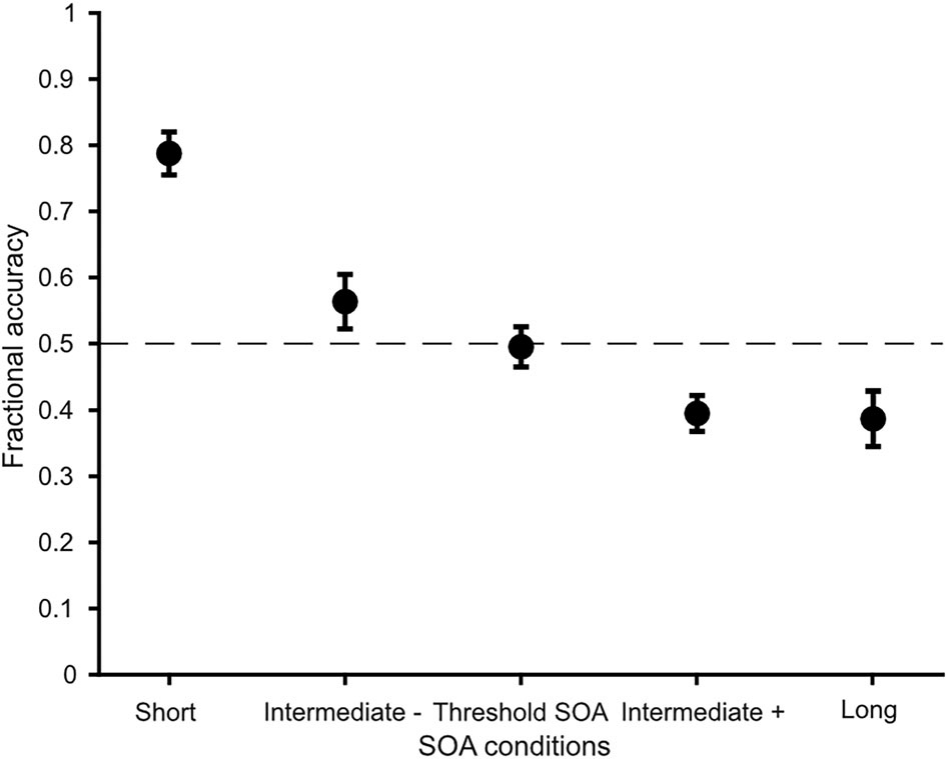
Behavioural performance. Fractional accuracy is plotted against the different SOA conditions. The dashed line marks the 50% mark indicating the performance level we expected to see for the threshold SOA. Data are presented as mean ± SEM. A one-way ANOVA reveals a significant effect of SOA conditions (*p* < 0.001).

In a staircase procedure prior to the experiment, we determined the SOA, at which participants were able to detect the empty position in 50% of the trials (60.0 ± 7.7 ms; mean ± SEM). The average threshold SOA was 60 ms (±38.45 ms; range, 16–144 ms). In the main experiment, we determined recognition rates as a function of different SOAs (short, threshold SOA; long, intermediate plus and minus SOAs; see Materials and Methods). Recognition rates were at 0.80 ± 0.03 (mean ± SEM) for short SOA; 0.55 ± 0.04 (mean ± SEM) for intermediate minus; 0.45 ± 0.03 (mean ± SEM) for threshold SOA; 0.42 ± 0.03 (mean ± SEM) for intermediate plus; and 0.31 ± 0.04 (mean ± SEM) for long SOA (Fig. 2). A one-way ANOVA revealed a significant main effect of SOAs (*F* = 20.9, *p* < 0.001; Fig. 2). Post hoc Tukey–Kramer tests showed that the short SOA differed significantly from all other SOAs (all pairwise-comparisons *p* < 0.001). The intermediate minus SOA differed significantly from the intermediate plus SOA (*p* = 0.013) and the long SOA (*p* = 0.004). Post hoc tests showed no significant differences between intermediate minus SOA and threshold SOA (*p* = 0.212), threshold SOA and intermediate plus SOA (*p* = 0.769), threshold SOA and long SOA (*p* = 0.564), and intermediate plus SOA and long SOA (*p* = 0.999).

### Phase contrasts

**Figure 3.**
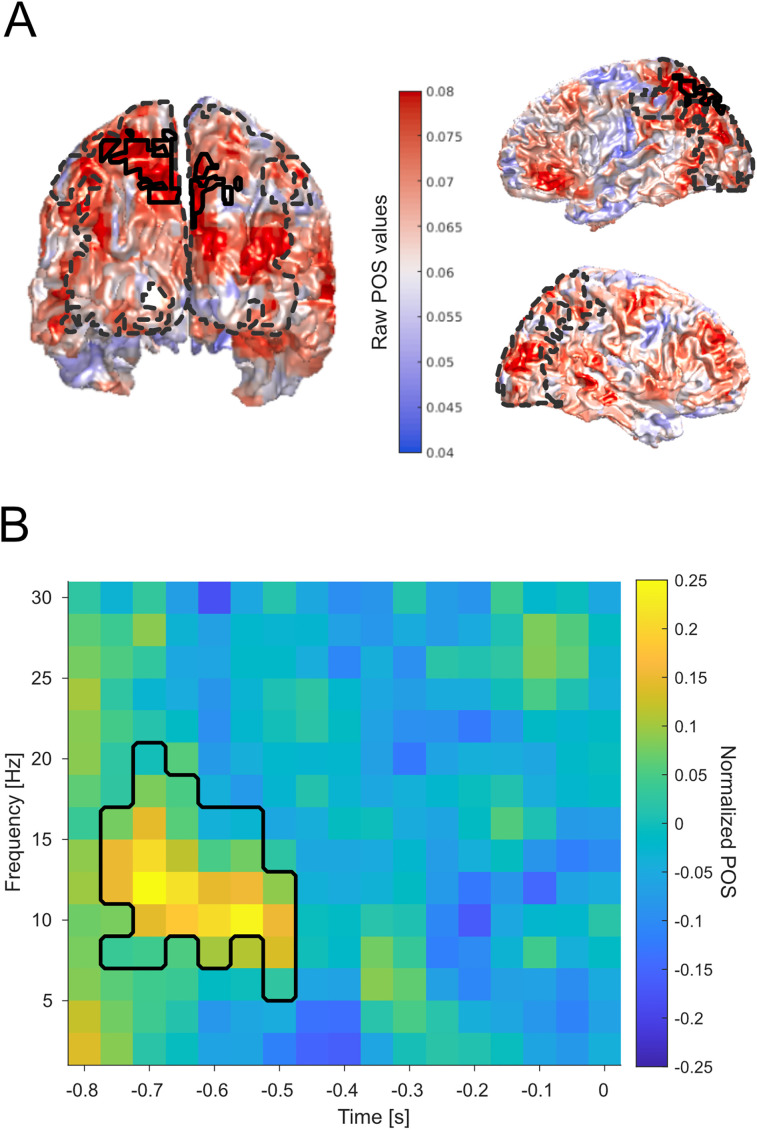
Phase opposition sum (POS) effects. ***A***, POS values projected on a template MNI brain viewed from the posterior (left) and right and left view (right). The black outline indicates the significant region/virtual channels (*p* = 0.048; summed *t* values, 2,722.4; *t* value range, 2.07–6.57). The dashed line indicates the region of interest for statistical comparison. ***B***, Averaged normalized phase opposition values over all significant virtual channels of the cluster (see panel ***A***). The black outline indicates the significant time–frequency range.

To investigate whether perception is influenced by the phase of neuronal oscillations, we split all trials with a threshold SOA according to participants' perception (i.e., correct and incorrect trials). Next, we analyzed the putative phase differences between correct and incorrect trials by computing the phase opposition sum (POS, VanRullen, 2016a) on a source level for each time–frequency grid sample and subsequently performing a cluster-based permutation test. The average trial count for correct trials was mean = 87.4 (±34.3 STD) and mean = 81.2 (±25.1) for incorrect trials. The results revealed a significant cluster (*p* = 0.048; summed *t* values, 2,722.4; *t* value range, 2.07–6.57) between −0.8 and −0.5 s and between 6 and 20 Hz, showing the most pronounced effect between 8 and 16 Hz (Fig. 3*B*). The significant cluster was located at the parietal lobe (Fig. 3*A*, left) and more prominent in the left hemisphere (Fig. 3*A*, right). We tested for systematic POS differences between high- and low-power trials, by recalculating the POS values for high- and low-power trials. We averaged the POS values per subject for the channel–time–frequency points we reported above. We found no power difference between POS values (*t* = −0.61, *p* = 0.577).

### Phase and behavioral performance

**Figure 4.**
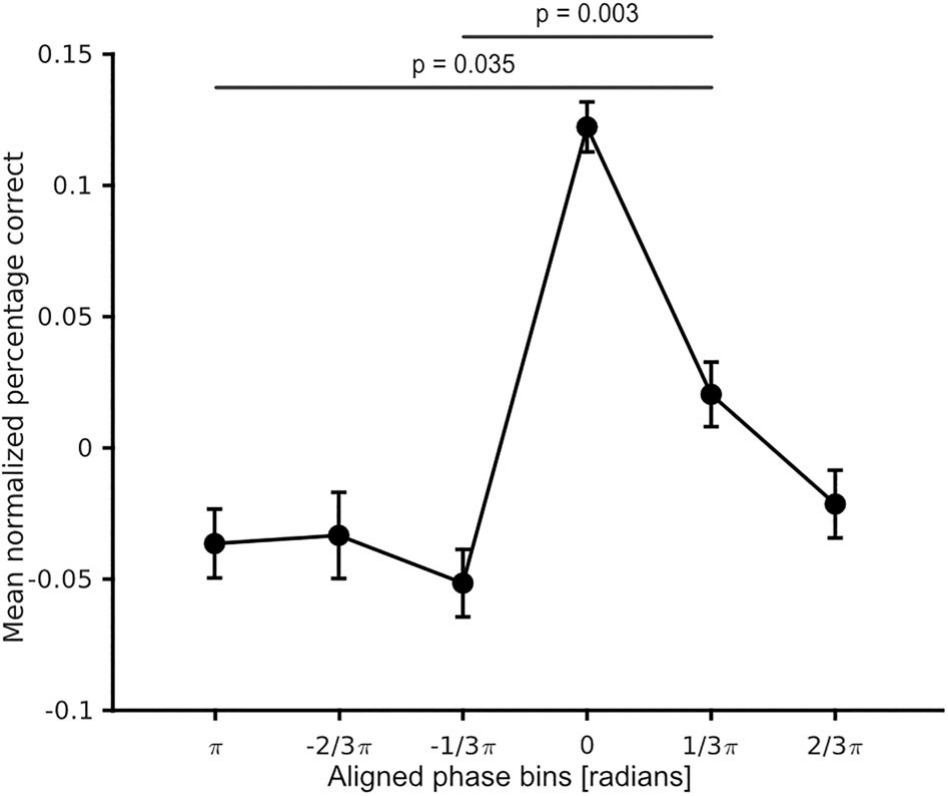
Phase dependence of behavior. Normalized fractional accuracy plotted against the binned prestimulus phase. Data are shown as mean ± SEM. An ANOVA revealed a significant effect of phase bins (*F* = 3.87, *p* = 0.005). The black lines indicate significant differences between phase bins in post hoc pairwise comparisons.

We investigated how behavior was modulated by the momentary phase. To this end, we computed for each participant the phase for all trials at the time–frequency sample showing the highest POS value. Next, we binned single trials according to their momentary phase in six bins and averaged for each bin participants' responses (Fig. 4). A one-way ANOVA excluding the phase bin containing the aligned highest responses (Bin 0) revealed a significant effect of phase bin on accuracy (*F* = 3.9, *p* = 0.005). A post hoc Tukey–Kramer test showed a significant difference between bin π and bin (1/3)π (*p* = 0.035) and between bin −(1/3)π and bin (1/3)π (*p* = 0.003) and a trend toward a significant difference between bin −(2/3)π and (1/3)π (*p* = 0.053). We additionally restricted the analysis to the alpha band (8–12 Hz), following the same pipeline but with a different frequency range of interest. The results showed no significant influence of phase on behavior when restricted to the alpha phase (*F* = 0.58, *p* = 0.681).

### Phase and ERF

**Figure 5.**
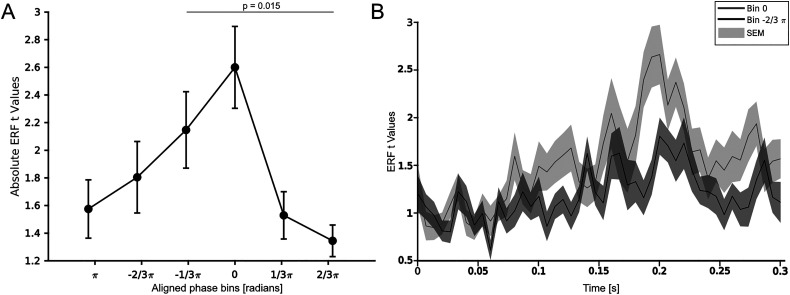
Phase dependence of ERF amplitude. ***A***, ERF *t* values plotted against the binned prestimulus phase. Data are shown as mean ± SEM. An ANOVA revealed a significant effect of phase bins (*F* = 3.4, *p* = 0.011). The black lines indicate significant differences between phase bins in post hoc pairwise comparisons. ***B***, ERF amplitude as absolute ERF *t* values are shown for two phase bins. Bin 0 in light gray and bin −(2/3)π in black. The shaded area is the standard error of the mean. No statistics were applied, only for visualization. See Extended Data Figure 5-1 for individual ERF data.

We binned trials according to their momentary phase in equally spaced phase bins. Next, we computed per participant the ERF *t* values for all trials in each bin and averaged the ERF *t* values across participants. The ERF *t* values were obtained by contrasting the baseline period (−0.8 to −0.5 s) with the poststimulus time period of interest (0–0.3 s). A one-way ANOVA showed a significant effect of phase bins (*F* = 3.4, *p* = 0.011; Fig. 5*A*). A post hoc Tukey–Kramer test showed a significant difference between bin −(1/3)π and bin (2/3)π (*p* = 0.016) and a trend toward a significance between bin −(1/3)π and bin (1/3)π (*p* = 0.064). Additionally, we tested if these effects were also found when restricted to the alpha band. We performed the same analysis, but this time we calculated the momentary phase between 8 and 12 Hz and not between 2 and 30 Hz, as we did before. Our additional analysis showed that there was no difference between phase bins when restricted to the alpha band (*F* = 1.98, *p* = 0.102). When visually inspecting the averaged ERF curves for two different phase bins, we saw a clear amplitude difference (Fig. 5*B*). Single subject data are shown as extended data (Extended Data Fig. 5-1). At last, our analysis of binning the ERF *t* values based on the phase values used for the behavioral data revealed the same qualitative pattern, but no significant effect of phase bin on ERF *t* values (*F* = 1.53, *p* = 0.199).

10.1523/ENEURO.0471-23.2024.f5-1 Figure 5-1 Individual ERF amplitude. Subplots show the individual ERF amplitude (black line) for each subject. Y-axis shows absolute ERF t-values and x-axis time in seconds. Grey lines indicate analysis window for N170 peak analysis. Download Figure 5-1, TIF file.

### Correlation of peak frequency and threshold SOA

**Figure 6.**
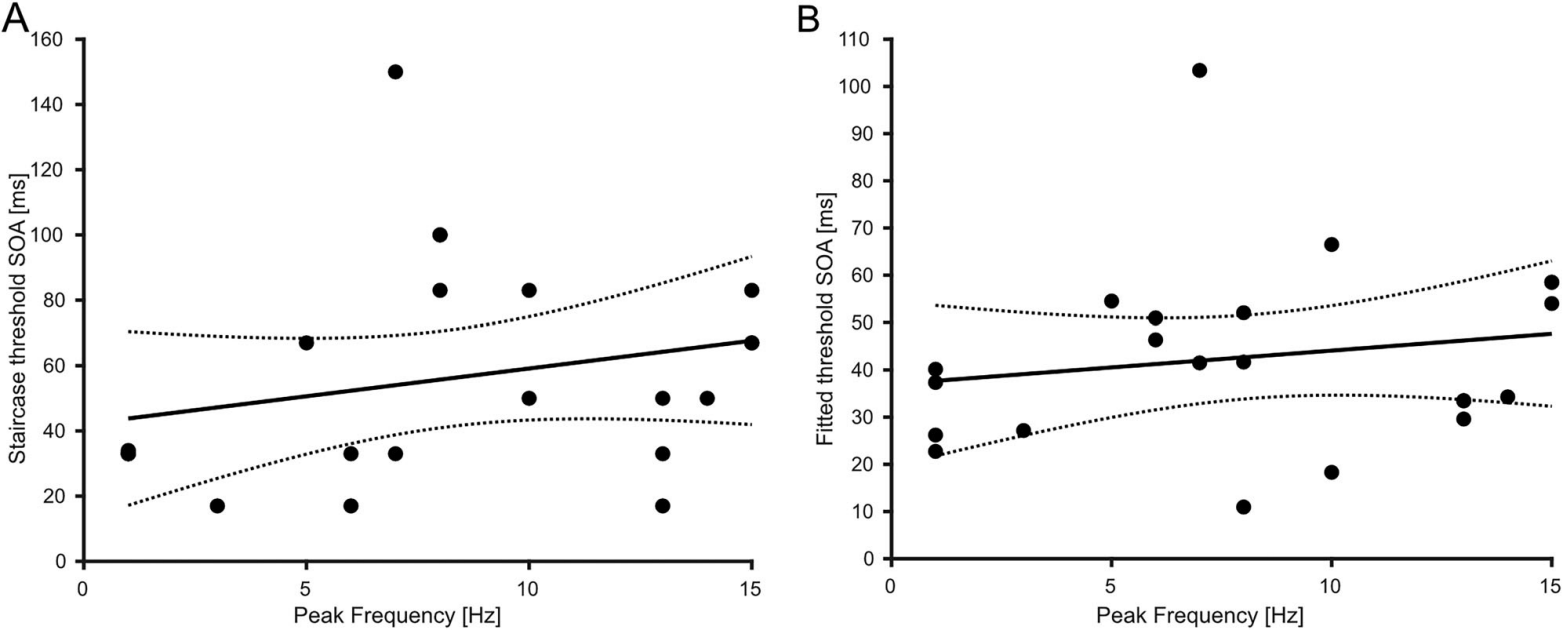
Correlation between peak frequency and threshold SOAs. ***A***, Threshold SOA determined prior to the experiment plotted against the peak frequency (see Materials and Methods for details). Pearson’s correlation revealed no significant correlation (*r* = 0.25, *p* = 0.26). ***B***, Same as panel ***A***, but this time correlation between estimated fitted threshold SOA and peak frequency (see Materials and Methods for details). Pearson’s correlation revealed no significant correlation (*r* = 0.18, *p* = 0.423). The black line represents the linear regression, and the dotted lines represent the 95% confidence intervals for the mean.

We investigated whether the individual peak frequency correlates with individual threshold SOA. Therefore, we correlated peak frequency with the estimated SOA prior to the experiment (staircase; Fig. 6*A*) and the fitted (Fig. 6*B*) threshold SOA (see Materials and Methods for details). For three participants, the fitted threshold SOA could not be determined reliably (*R*^2 ^< 0.33). These participants had to be excluded from the respective correlation analysis. We did not find any significant correlation between individual peak frequencies and staircase threshold SOAs (*r* = 0.25, *p* = 0.26) or between peak frequencies and fitted threshold SOAs (*r* = 0.18, *p* = 0.43). As for our supplementary analysis, where we correlated power peak frequency with the threshold and fitted threshold SOA, we found no significant correlation between either staircase (*r* = −0.1, *p* = 0.64) or the fitted threshold SOAs (*r* = −0.24, *p* = 0.24). Lastly, we restricted the frequency range of interest of our initial analysis to the alpha band of 8–12 Hz, and this restriction did not change the results. Neither for the staircase threshold SOA (*r* = 0.25, *p* = 0.26) nor for the fitted threshold SOA (*r* = 0.18, *p* = 0.43) was a significant correlation found for the peak frequency.

## Discussion

There is an ongoing debate whether prestimulus phase of neuronal oscillations influences perception (Keitel et al., 2022; Harris, 2023). Most evidence favoring a crucial role of phase stems from visual detection tasks. Here, we investigated how prestimulus phase modulates visual temporal integration. We found phase differences when comparing correct with incorrect trials. These phase effects were in the frequency range between 6 and 20 Hz and −0.8 and −0.5 s prior to stimulus presentation, mainly in parieto-occipital areas. Additionally, accuracy decreased when deviating from the preferred prestimulus phase. Finally, prestimulus phase modulates poststimulus visual processing as the N170 amplitude attenuated when deviating from the individually preferred phase. However, we found no evidence that the individual frequency of the prestimulus phase correlates with the temporal integration threshold.

Previous studies investigating visual detection tasks reported phase effects in the alpha band (Busch et al., 2009; Mathewson et al., 2009; Landau and Fries, 2012; Kraut and Albrecht, 2022; Zazio et al., 2022). In our study, the effects are largest in the range of 8–16 Hz but also exceed the low beta band (20 Hz). It remains an open question whether these broadband effects stem from averaging interindividual differences or represent a genuine broadband effect. Visual inspection of the results on a single subject level could not give a clear answer in either direction. Thus, our analysis seems not suited to investigate this broadband effect further. However, the higher frequencies in our study might depend on task demands. It has been reported that alpha peak frequency shifts to higher frequency in cognitively more demanding tasks (Haegens et al., 2014). Similarly, studies have shown that occipital alpha peak frequencies differ between visual integration or segregation tasks (Wutz et al., 2018; Sharp et al., 2022; Han et al., 2023). Relevant frequency shifted with task difficulty also in a visual search task (Merholz et al., 2022). In line with these findings, the higher frequencies here reported might be explained by the higher task difficulty of a temporal discrimination task compared with detection tasks. On the other hand, Wutz et al. found differences in peak frequencies between the integration and segregation tasks. They found a slightly lower peak frequency for the integration task compared with the segregation task (Wutz et al., 2018). It could be that the task demand was slightly higher for the segregation task compared with the integration task (see slightly lower maximum recognition rates). While most studies report phase effects over the occipital lobe (Wutz et al., 2018; Alexander et al., 2020; Fakche et al., 2022; Zazio et al., 2022), we found phase effects in the parietal lobe. One possible explanation could be that higher-order visual areas are involved in solving this task. A classical hypothesis states that the “where” pathway—which processes information about the spatial location of objects—includes the parietal lobe (Mishkin et al., 1983). A potential reason for effects in the parietal lobe might be that these areas are engaged in our spatial integration and detection task.

The reported performance effects (decreasing when deviating from the preferred phase) are smaller compared with other studies (∼7% difference between highest and lowest bin compared with ∼13%; Busch et al., 2009; Dugue et al., 2011; Baumgarten et al., 2015; Fakche et al., 2022). Task difficulty could be a leading cause for the difference, while the effect pattern is comparable. In addition, we found that prestimulus phase modulates the amplitude of the poststimulus N170 component. In line with our finding, prestimulus phase has been reported to modulate global field potential (Busch and VanRullen, 2010; Dou et al., 2022; note that Dou et al. found these effects only for high-power trials), TMS-evoked ERPs (Romei et al., 2012; Fakche et al., 2022), and visual awareness negativity (Krasich et al., 2022). In sum, these studies suggest across a variety of different tasks that prestimulus phase can modulate poststimulus components. However, we found that the prestimulus phases in different, but overlapping areas modulate behavior and the amplitude of the N170 component. For the behavioral data, we looked for prestimulus phase values of the highest POS values, and for the ERF data, we searched for the phase values of the 100 channels showing the highest ERF amplitude. It could be that modulation of behavior and ERF amplitude happen in separated areas.

While our results are comparable to detection task results, the underlying mechanism is not well understood. Earlier studies proposed the idea that stimulus presentation triggers a phase shifted toward a preferred phase, inducing a phase reset (Makeig et al., 2002). When prestimulus phase is at the preferred phase, stimulus processing is optimal, leading to increased evoked responses and improved performance. Whereas, when the phase has to shift toward the preferred phase, processing is less optimal, leading to decreased evoked responses and behavior. This could be investigated by comparing phase angle values before and after phase reset. Such analyses, however, might be practically less straightforward due to potential temporal smearing effects by the methods for phase analysis (Brüers and VanRullen, 2017).

Neuronal phase might also indicate the current excitability of the underlying neuronal population (Bishop, 1932; Lakatos et al., 2007; Fakche et al., 2022). In theory, cortex excitability changes rhythmically with phase, while excitability is highest at a preferred phase (Mazaheri and Jensen, 2006; Jensen and Mazaheri, 2010; Schaworonkow et al., 2019). Neuronal processing would be facilitated at such a preferred phase and putatively lead to higher ERPs. Stimulus processing would be more effective and putatively lead to better behavioral performance. Cortical excitability has also been repeatedly linked to the power of alpha oscillations (Romei et al., 2008; Lange et al., 2013; Dou et al., 2022). Here, low alpha power is beneficial for perception, while high alpha power suppresses excitability. Notably, phase effects on behavior and neuronal processing have often been reported for high but not for low alpha power trials (Mathewson et al., 2009; Alexander et al., 2020; Dou et al., 2022; Fakche et al., 2022; Ozdemir et al., 2022). The reason could be that, when power is low, cortical excitability is high and phase effects are less notable or irrelevant. In contrast, when power is high, excitability is low, and phase matters more strongly as excitability differs more strongly between different phases.

Between studies, there is a high variability regarding the latency of the reported phase effects. Some report effects close to stimulus onset (∼100–200 ms before stimulus onset; Alexander et al., 2020; Fakche et al., 2022; Zazio et al., 2022), while other studies—including the present study—report phase effects several 100 ms before stimulus onset (Hanslmayr et al., 2013; Baumgarten et al., 2015; Kraut and Albrecht, 2022). We can only speculate about the causes of these discrepancies. One reason might be that the timing of effects depends on the task (see above discussion on the frequency band). Temporal smearing effects of peri- or poststimulus effects into the prestimulus period (Brüers and VanRullen, 2017) might also overshadow putative phase effects close to stimulus presentation. Another explanation could be that slow frequencies orchestrate faster frequencies. The evidence stems from the reported power-phase coupling between higher and lower frequencies (Palva, 2005; Canolty et al., 2006; Axmacher et al., 2010; Voytek, 2010). Similarly, studies have reported phase–phase coupling of neuronal oscillations in different frequency bands (Belluscio et al., 2012; Scheffer-Teixeira and Tort, 2016). Underlying (ultra-)slow oscillation orchestrating alpha activity on a network level would lead to a rhythmic occurrence of phase effects, with a visible component several milliseconds before stimulus presentation. Respiration could be such a slow oscillation, by increasing the overall oxygen level, which would lead to higher excitability. Respiration has an effect on behavioral measures (Johannknecht and Kayser, 2022) and modulates alpha power at rest and during detection tasks (Kluger et al., 2021). In addition, the slow cardiac cycle influences neuronal activity and behavioral performance rhythmically (Kim et al., 2019; Al et al., 2020). Such slow fluctuations have also been reported for perception (Monto et al., 2008; Palva and Palva, 2012). By investigating longer prestimulus periods of several seconds, future studies might shed more light on such rhythmic patterns and how they affect phase and other components of neuronal oscillations.

Finally, we investigated if a specific frequency band is relevant for unimodal visual temporal integration. Recent studies found evidence for a correlation between individual alpha peak frequencies and behavioral performance in visual or multimodal studies (Samaha and Postle, 2015; Keil and Senkowski, 2017; Minami and Amano, 2017; Bastiaansen et al., 2020; Migliorati et al., 2020; Venskus and Hughes, 2021; Venskus et al., 2021). A common interpretation of such correlations is that the cycle of a neuronal oscillation might reflect temporal integration windows, while the frequency shapes the window size. Following a slightly different approach, by taking the frequency with the highest individual phase effect (Baumgarten et al., 2015), we found no evidence of an influence of frequencies on perception or temporal integration.

While several studies reported an effect of phase on perception in different tasks, interpretations about the functional role of phase differ. In short, two hypotheses exist: The hypothesis of rhythmic perception states that the intensity or probability of perception changes rhythmically with phase (VanRullen and Koch, 2003; VanRullen, 2016b). The hypothesis of discrete perception states that the phase relative to stimuli determines whether the stimuli fall within the same or different perceptual cycles leading to different perceptions (VanRullen and Koch, 2003; Baumgarten et al., 2015). Our results show that phase correlates with behavior and poststimulus ERFs, while we found no support for perceptual cycles. Therefore, we suggest that unimodal visual temporal integration is a rhythmic process, in a wider frequency range as expected, and potentially even modulated by even slower underlying oscillations.

A limitation of this study is that the estimation of individual phase effects is typically less stable than determining peak frequencies. Such noisy estimation might influence the results. In addition, we observed high subject variability in the behavioral data and phase opposition effects (absolute value, timing, frequency). Our analysis captures the overlapping effects on a group level. Other study designs would be more suited to capture individual differences.

In addition, it should be noted that several studies report null findings with respect to phase effects on perception or neuronal activity (Ruzzoli et al., 2019; Michail et al., 2022; Zazio et al., 2022; Melcón et al., 2024). Here, we can only speculate about the discrepancies. In our study, phase effects were very local in space (Palva, 2005; Baumgarten et al., 2015). In addition, typically phase effects are smaller than power effects. Therefore, phase effects might be too small to be detected or overshadowed by other effects, especially when analyzed on a sensor level, as spatial leakage of other effects might be stronger than the actual phase effect. It also seems that phase effects are only visible in tasks near the perceptual threshold, but not for easier tasks. It remains to be studied whether phase plays a continuous role in our perception or is only relevant near the perceptual threshold. Finally, potential phase effects might be overlooked due to methodological problems in the analyses (Harris, 2023).

In conclusion, we found an effect of phase of alpha oscillations in a visual temporal discrimination task and on neuronal processing. These results suggest that perception is a rhythmic process and/or that the phase modulates stimulus processing. While these results are of correlative nature, future studies might investigate causal relationships between phase and perception/neuronal processing. To test a putative causal relationship, phase resets could be triggered externally and the consequences on behavior can be tested.

## Synthesis

Reviewing Editor: Ifat Levy, Yale School of Medicine Decisions are customarily a result of the Reviewing Editor and the peer reviewers coming together and discussing their recommendations until a consensus is reached. When revisions are invited, a fact-based synthesis statement explaining their decision and outlining what is needed to prepare a revision will be listed below. The following reviewer(s) agreed to reveal their identity: Philipp Ruhnau, Laetitia Grabot. Note: If this manuscript was transferred from JNeurosci and a decision was made to accept the manuscript without peer review, a brief statement to this effect will instead be what is listed below.

This study investigated whether prestimulus EEG phase impacts temporal integration. The authors used a visual discrimination task where integration of two presented stimuli favors correct perception. This complements existing literature which investigated this question with detection or simultaneity judgement tasks, but not with a task requiring temporal integration. The authors found that prestimulus phase between 6 and 20Hz differs between correct and incorrect responses and modulates the evoked response (N170).

This is a thorough revision of a complex and interesting study. The authors used a relevant task to study visual integration and proper statistical methods to assess their results, and their responses to previous reviewers were convincing. The manuscript would gain clarity, however, by explicitly articulating in the introduction the specific gap that is being addressed. Additionally, delving further into the inquiry of broadband versus narrow-band effects would be advantageous for the manuscript: this can be achieved by showing individual plots of phase opposition effects and by additional narrow-band analyses. Below are the specific comments:

Main points

- The introduction could be more explicit about how this study specifically tackled the question of the functional role of phase in temporal perception. There are some missing references of former studies tackling this question (Milton &Pleydell-Pearce, 2016; Wutz, Melcher, Samaha, 2018). What was the added value of this study compared to the 2018 study from Wutz and colleagues, which used a similar task with MEG recordings?

- Regarding the POS results, it would be great to gain a better understanding of the origin of the broadband frequencies. Is this broadband cluster only generated by inter-individual differences of narrow-band clusters, artificially generated by averaging, or is it a true broadband effect? Could the authors show individual POS to answer this question?

- The authors did not find any significant correlation between the SOA leading to 50% of correct responses and the maximal POS frequency. This might not be surprising given that the individual frequencies cover a large range, such that it is possible that different oscillations with distinct functional roles are compared together. The paper would be strengthened if the authors conduct the phase analysis (fig 4) and the correlation analysis (fig 5) in a more hypothesis-driven approach, by restricting the frequency band of interest to the alpha band. It will be interesting to see if the correlations from Wutz et al (2018) are replicated or not, and can lead to an interesting discussion on narrow vs broadband effects.

Minor points

- Could the authors show a time course of the ERF, for two opposed phases for instance, to visualize the phase effect shown in Fig 5 (for instance at bin 2/3pi or pi vs 0)? Could you also show the individual ERFs and the extracted peak for each individual, to check that the N170 peaks were correctly identified (as a supplementary figure)?

- In the discussion, the authors propose that the frequency range found in this study to impact visual perception is due to increased task demands compared to detection tasks. Reviewers suggested that the crucial aspect may not merely be the higher overall frequency, but rather the broadband frequency range (spanning alpha and beta bands as noted by the authors). How would the authors interpret this broadband effect regarding task demands? Additionally, this explanation may be at odds with Wutz et al (2018)'s result: they found decreased alpha frequency in the integration task used here, compared to their segregation task.

- When the authors speculate about the underlying mechanism behind phase effect, could they discuss their results with respect to the perceptual cycles framework (e.g. Van Rullen, 2016, Perceptual cycles)? The idea is that a preferred phase would allow the two visual stimuli to fall within the same perceptual cycle, leading to increased integration, favoring correct responses. Can the authors elaborate on how their data align with (or deviate from) this framework?

- Could the authors add some discussion about the source localization result - in the parietal area?

- p3, l66: What was the screen refresh rate?

- p3, l88: the step of the staircase procedure is not given.

- p6, l220: the selected grid points likely come from the source estimate. Are they selected from the whole brain or from the restricted ROIs?

- p7, l237: There are repetitions and redundancy in the text.

- p7, l246: the "fitted SOA" is also based on experimental data. This phrasing is confusing. Maybe you could say that the initial SOA is based on data from the staircase procedure, while the fitted SOA comes from the data recorded during MEG acquisition (staircase SOA vs fitted SOA).

- p8, l265: Could you give the range of values found as the threshold SOA, to give an idea of the inter-individual variability?

- p8, l289: "We tested for systematic power differences between high and low power trials"-> Typo (should be corrected into "we tested for systematic POS differences between high and low power trials").

- p8, l294: was the phase binning analysis done on all trials (as written), or only on threshold SOA conditions?

- S1: The plot for subject S26 is missing.

- significance statement: insides --> insights; also the second sentence seems incomplete, probably good to revise.

- Line 194: an --> a

- Line 299: please avoid interpreting strength of significance ranges ('strong trend towards significance' --> trend towards significance)

- Figure 3 caption: this states significant effect and reports p=0.48 - is this a typo?

- Reference list and citation: The Zazio reference is listed twice (once the online version and once the version with final issue/page numbers). Ideally, these two are merged to the final version

## Author Response

Reply to Reviewer Comments We thank the reviewer for their feedback and suggestions to further improve our manuscript. We will address the comments in a point-by-point replay, citing reviewer comments in bold, our reply in normal font and revised text in the manuscript in italic.

Please include a statement in the Materials and Methods section, under the heading "Code Accessibility", indicating whether and how the analysis code can be accessed, including any accession numbers or restrictions, as well as the type of computer and operating system used to run the code We added an accessibility statement at the end of the methods sections. Please see p.7 II 270-272. for this.

Main points The introduction could be more explicit about how this study specifically tackled the question of the functional role of phase in temporal perception. There are some missing references of former studies tackling this question (Milton &Pleydell-Pearce, 2016; Wutz, Melcher, Samaha, 2018). What was the added value of this study compared to the 2018 study from Wutz and colleagues, which used a similar task with MEG recordings? We added the references and added a few additional clarifications what our study adds to the overall discourse. p. 2 II 52-59: "While most studies focused on the role of phase for detection, few studies investigated the role of phase for temporal integration of visual stimuli. Wutz et al. used a similar task as we used in the present study to investigate visual temporal integration. Instead of analysing phase, however, they focused on the functional role of frequency for visual integration (Wutz et al., 2018). In addition, Milton &Pleydell-Pearce (2016) found that prestimulus phase correlated with performance in a temporal simultaneity task. In the present study, we expand these finding by determining whether the prestimulus phase influences temporal integration mechanisms by modulating early poststimulus sensory processing or whether the frequency of prestimulus phase effects determines temporal integration windows." Regarding the POS results, it would be great to gain a better understanding of the origin of the broadband frequencies. Is this broadband cluster only generated by inter-individual differences of narrow-band clusters, artificially generated by averaging, or is it a true broadband effect? Could the authors show individual POS to answer this question? The visual inspection of the data on single-subject level did not give a clear answer. On the one hand, we do not see on single-subject broadband effects similar to the group-level effect. On the other hand, single-subject data do not show clearly defined narrowband effects. In sum, it remains uncertain whether inter-individual differences are causing the broad-band effect or if it is indeed a genuine broadband phenomenon. We added a comment into the discussion. p. 10 II 358-361: "It remains an open question whether these broadband effects stem from averaging inter-individual differences or represent a genuine broadband effect. Visual inspection of the results on single subject level, could not give a clear answer in either direction. Thus, our analysis seems not suited to investigate this broadband effect further." The authors did not find any significant correlation between the SOA leading to 50% of correct responses and the maximal POS frequency. This might not be surprising given that the individual frequencies cover a large range, such that it is possible that different oscillations with distinct functional roles are compared together. The paper would be strengthened if the authors conduct the phase analysis (fig 4) and the correlation analysis (fig 5) in a more hypothesis-driven approach, by restricting the frequency band of interest to the alpha band. It will be interesting to see if the correlations from Wutz et al (2018) are replicated or not, and can lead to an interesting discussion on narrow vs broadband effects.

We rerun our analysis, restricted only to the alpha range (8 - 12 Hz) and - again - found no significant effects for the phase or correlation analysis. We added the additional analysis in the manuscript. p. 8/9 II 316-318: "We additionally restricted the analysis to the alpha band (8-12 Hz), but following the same pipeline as above. The results showed no significant influence of phase on behaviour, when restricted to the alpha phase (F = 0.58, p = 0.681)." p. 9 II 324-328: "Additionally, we tested if these effects were also found, when restricted to the alpha band. The same analysis was performed but this time, choosing ERF values between 8 - 12 Hz and averaging over these frequency points. Our additional analysis showed no difference between phase bins when restricted to the alpha band (F = 1.98, p = 0.102)." Minor points Could the authors show a time course of the ERF, for two opposed phases for instance, to visualize the phase effect shown in Fig 5 (for instance at bin 2/3pi or pi vs 0)? Could you also show the individual ERFs and the extracted peak for each individual, to check that the N170 peaks were correctly identified (as a supplementary figure)? We inspected the difference of the ERFs for different phase bins and saw clear differences in amplitude. We would like to focus on the main results and would therefore no include additional figures. eNeuro permits the use of supplementary material therefore we included a small statement in the results. p.9 II 328-329: "When visually inspecting the averaged ERF curves for three different phase bin, we saw a clear amplitude difference." In the discussion, the authors propose that the frequency range found in this study to impact visual perception is due to increased task demands compared to detection tasks. Reviewers suggested that the crucial aspect may not merely be the higher overall frequency, but rather the broadband frequency range (spanning alpha and beta bands as noted by the authors). How would the authors interpret this broadband effect regarding task demands? Additionally, this explanation may be at odds with Wutz et al (2018)'s result: they found decreased alpha frequency in the integration task used here, compared to their segregation task.

We have added an additional paragraph to the Discussion section, which discusses the question regarding integration/seggregation. As discussed above (#2), our additional analysis did not give a clear answer regarding the origin of the broadband effect. Therefore, we feel that a discussion about a potential link between putative broadband effects and task demands would be too speculative. p.10 II 367-371: "On the other hand, Wutz et al. found the opposite effect that alpha frequency decreased in their integration task compared to their segregation task (Wutz et al., 2018). A crucial difference in study design is, that participant had to change strategies during the experiment. It could be that due to the switch the difference in alpha peak frequency occurred." When the authors speculate about the underlying mechanism behind phase effect, could they discuss their results with respect to the perceptual cycles framework (e.g. Van Rullen, 2016, Perceptual cycles)? The idea is that a preferred phase would allow the two visual stimuli to fall within the same perceptual cycle, leading to increased integration, favoring correct responses. Can the authors elaborate on how their data align with (or deviate from) this framework? We now discuss our results in the context of perceptual cycles. p.12 II 442-451: "While several studies reported an effect of phase on perception in different tasks, interpretations about the functional role of phase differ. In short, two hypotheses exist: the hypothesis of rhythmic perception states that the intensity or probability of perception changes rhythmically with phase. The hypothesis of discrete perception states that the phase relative to stimuli determines whether the stimuli fall within the same or different perceptual cycles leading to different perceptions (VanRullen 2016). Our results show that phase correlates with behaviour and post-stimulus ERFs, while we found no support for perceptual cycles. Therefore we suggest that unimodal visual temporal integration is a rhythmic process, in a wider frequency range as expected, and potentially even modulated by even slower underlying oscillations." Could the authors add some discussion about the source localization result - in the parietal area? We now add a discussion about the parietal areas. p.10 II 371-377: "While most studies report phase effects over the occipital lobe (Wutz et al., 2018; Alexander et al., 2020; Fakche et al., 2022; Zazio et al., 2022), we found phase effects in the parietal lobe. One possible explanation could be that higher order visual areas are involved in solving this task. A classical hypothesis states that the "where" pathway - which processes information about the spatial location of objects - includes the parietal lobe (Mishkin et al., 1983). A potential reason for effects in the parietal lobe might be that these areas are engaged in our spatial integration and detection task." p3, l66: What was the screen refresh rate? We used a projector system with a translucence screen. The projector had a refreshment rate of 60 Hz. p.3 II 88-89: "We projected the stimuli on a translucence screen using a projector located outside the magnetically shielded room (Panasonic, PT-DW700E; 60 Hz refresh rate) and a mirror system." p3, l88: the step of the staircase procedure is not given.

We added this information to the manuscript. p.3 II 95-96: "The step size of the increase/decrease was 16 ms (e.g. one frame)." p6, l220: the selected grid points likely come from the source estimate. Are they selected from the whole brain or from the restricted ROIs? We restricted our initial source estimate to our ROI, as described in the section "Source projection of MEG data" (p.4/5 II 140-154). We selected all grid points from this ROI.

We added clarification to the manuscript. p.4/5 II 149-150: "We restricted from here further analyses to regions of interest (ROI), defined by the AAL atlas (Tzourio-Mazoyer et al., 2002) implemented in FieldTrip." p7, l237: There are repetitions and redundancy in the text.

We removed the repetitions from the text. p7, l246: the "fitted SOA" is also based on experimental data. This phrasing is confusing. Maybe you could say that the initial SOA is based on data from the staircase procedure, while the fitted SOA comes from the data recorded during MEG acquisition (staircase SOA vs fitted SOA).

We changed the label of the experimental threshold SOA to staircase threshold SOA.

Please see p.7 II 247-268, p. 9 II 331-343. and p.18 II 679-685 for the changes. p8, l265: Could you give the range of values found as the threshold SOA, to give an idea of the inter-individual variability? We added this information to the manuscript. p.8 II 281-282: "The average threshold SOA was 60 ms ({plus minus} 38.45 ms, range 16-144 ms)." p8, l289: "We tested for systematic power differences between high and low power trials"-> Typo (should be corrected into "we tested for systematic POS differences between high and low power trials").

We corrected this typo. p.8 II 304-305: "We tested for systematic POS differences between high and low power trials, by recalculating the POS values for high and low power trials." p8, l294: was the phase binning analysis done on all trials (as written), or only on threshold SOA conditions? We choose only threshold SOA trials. We clarified this for both phase binning analysis. p.6 II 214-215: "For this time-frequency sample, we computed the momentary phase for each threshold SOA trial (see above for the parameters)." p. 7 II 234-236: "Next, from these 100 selected channels, we computed for each participant the momentary phase for the threshold SOA trials." S1: The plot for subject S26 is missing.

Thank you for pointing out the inconsistency, there was a typo in the methods section. We recorded 25 participants and fixed the error. p.3 II 66: "We recruited 25 participants (17 female, mean age 25.4 {plus minus} 5.1 years [SD]) for this study." Significance statement: insides --> insights; also the second sentence seems incomplete, probably good to revise.

We revised the significant statement.

Line 194: an --> a We removed this typo.

Line 299: please avoid interpreting strength of significance ranges ('strong trend towards significance' --> trend towards significance) We rephrased the sentences. p.8 II 315-316: "[...] and a trend towards significance difference between bin -2/3 π and 1/3 π (p = 0.053)." Figure 3 caption: this states significant effect and reports p=0.48 - is this a typo? We fixed the typo. p.18 II 666-667: "The black outline indicates the significant region/virtual channels (p = 0.048, summed t-values: 2722.4, t-value range: [2.07 6.57]." Reference list and citation: The Zazio reference is listed twice (once the online version and once the version with final issue/page numbers). Ideally, these two are merged to the final version.

Thank you for pointing that out. We merged the two references.
