# Supplementary figures and images for: Prestimulus Alpha Phase Modulates Visual Temporal Integration
Source: eNeuro. 2024 Sep 12;11(9):ENEURO.0471-23.2024. doi: 10.1523/ENEURO.0471-23.2024 (PMC11397504; doi:10.1523/ENEURO.0471-23.2024)

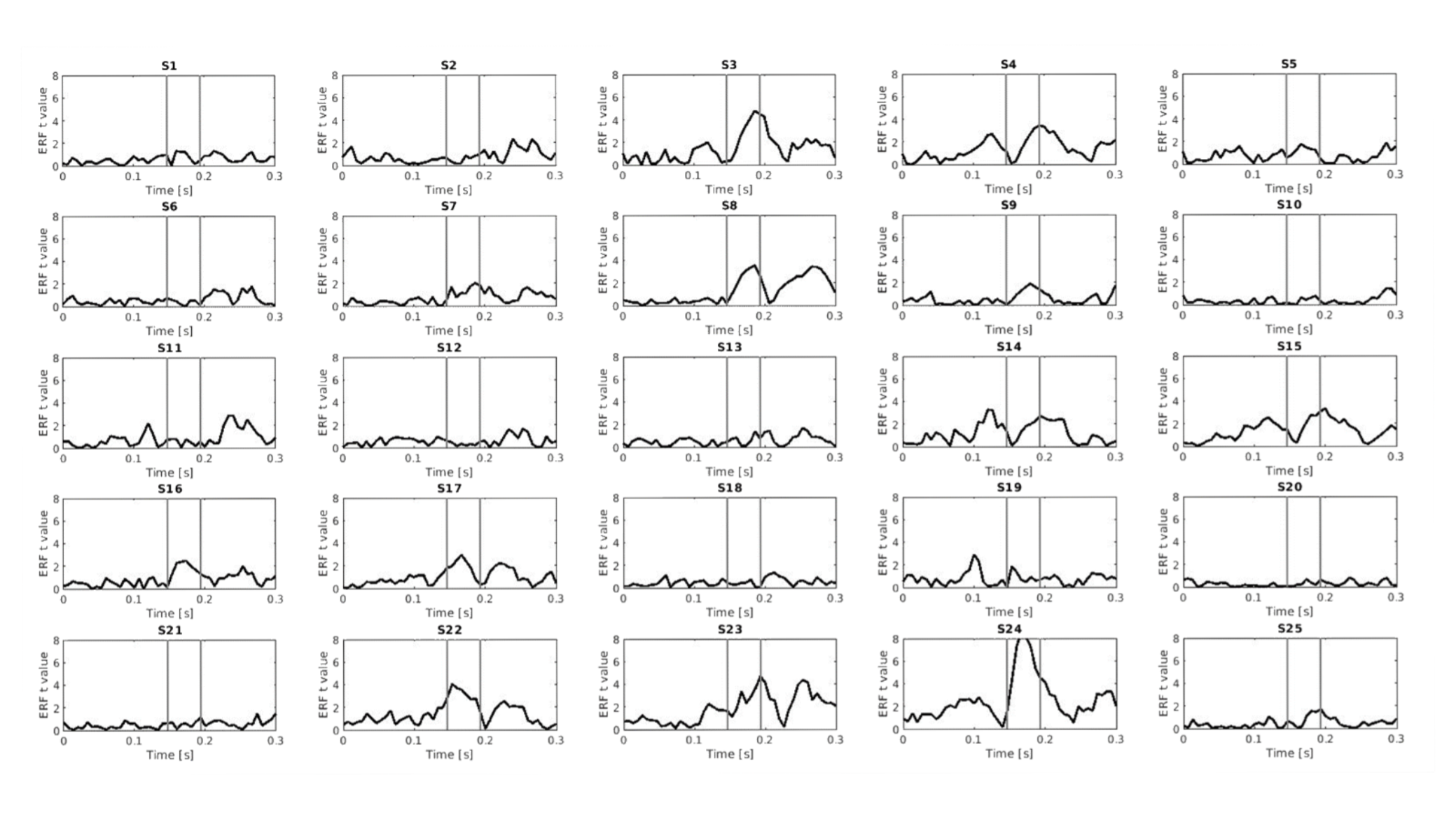

Supplement: Figure 5-1 — Individual ERF amplitude. Subplots show the individual ERF amplitude (black line) for each subject. Y-axis shows absolute ERF t-values and x-axis time in seconds. Grey lines indicate analysis window for N170 peak analysis. Download Figure 5-1, TIF file. [file eneuro-11-ENEURO.0471-23.2024-s001.tif]
